# Dynamic Changes in Community Deprivation of Access to Urban Green Spaces by Multiple Transport Modes

**DOI:** 10.3389/fpubh.2021.615432

**Published:** 2021-07-27

**Authors:** Shunqi Cheng, Zhiqiang Liao, Yu Zhu

**Affiliations:** ^1^Institute of Geography, Fujian Normal University, Fuzhou, China; ^2^Fujian Mapping Institute, Fuzhou, China; ^3^Asian Demographic Research Institute, Shanghai University, Shanghai, China; ^4^State Key Laboratory for Subtropical Mountain Ecology of the Ministry of Science and Technology and Fujian Province, Fujian Normal University, Fuzhou, China

**Keywords:** urban green spaces, dynamic changes, deprivation, multiple transportation modes, distribution disparity

## Abstract

Urban green spaces (UGSs) improve the quality of life of urban inhabitants. With the acceleration of urbanization and changes in traffic networks, it remains unclear whether changes in the distribution of UGSs can satisfy the needs of all inhabitants and offer equal services to inhabitants from different socioeconomic backgrounds. This study addresses this issue by analyzing dynamic changes in UGS accessibility in 2012, 2016, and 2020 for inhabitants of the central urban area of Fuzhou in China at the community level. The study introduces multiple transportation modes for an accessibility estimation based on a framework using the two-step floating catchment area method and examines the dynamic changes in community deprivation of UGS accessibility using Kernel regularized least squares, a machine learning algorithm. The results demonstrate that spatial disparities of UGS accessibility exist among the multi-transport modes and vary with time. Communities with high accessibility to UGSs by walking are scattered around the urban area; for accessibility by cycling, the high accessibility regions expand and surround the regions with low accessibility in the core urban areas, forming a semi-enclosed spatial pattern. However, the core urban spatial orientation of UGS accessibility by public transit demonstrates a reverse trend to the above two modes. The spatial pattern of UGS accessibility also varies over time, and the growth rate of accessibility slightly declined during the study period. Furthermore, the increase in UGS accessibility tended to slow from 2016–2020 compared with 2012–2016, and the trend toward equality was also erratic. The degree of deprivation for communities first weakened and was then aggravated, corresponding to the slowdown in the growth rate of accessibility, leading to the persistence existence of social inequality. Moreover, significant deprivation mainly exists among less educated people or those using the cycling and integrated travel modes. Although public transport is developing, deprived communities, such as communities with large proportion of older people, have experienced a decline in access to UGSs by public transport. Based on these findings, the study proposes a policy framework for the balanced distribution of UGSs as part of urbanization.

## Introduction

Urban green spaces (UGSs) primarily comprise urban land with naturally occurring and introduced vegetation. Such areas include lands used for greening within the scope of urban development land and green areas outside urban development land that positively affect urban ecology, landscape, and inhabitants' leisure life (1). UGSs provide a range of ecosystem services, such as beautifying the urban landscape, air purification, noise reduction, and local climate regulation, and contribute to maintaining urban biodiversity (2, 3). Meanwhile, UGSs protect the public health of urban inhabitants and provide a public space for leisure and recreation, which helps inhabitants maintain good social relations and improves their quality of life (4). These factors have great significance for improving inhabitants' well-being and urban sustainability (5, 6). Many studies have confirmed that inhabitants living near UGSs have more opportunities for physical activity, thus reducing the risk of obesity, alleviating mental stress, and improving mental health (7–10). As a critical public service component of any community, UGSs provide social and economic benefits by improving quality of life (11) and enhancing the economic vitality of the surrounding areas (12). The impact of UGSs on inhabitants' well-being is largely determined by accessibility. Combining the above benefits, studying inhabitants' dynamic changes in access to UGSs over time, and understanding the spatial distribution of UGSs and reasons for the social differential in utilizing them could greatly promote social equity.

Reasonable accessibility, which means that many people have access to UGSs services, is a precondition to realizing the value of UGSs (13, 14). However, rapid urbanization poses a large threat to the connection between humans and the natural environment, resulting in a series of conflicts between urban construction land development and the sufficient and sustainable provision of UGSs for inhabitants. This also leads to the problem of certain groups unable to use these UGSs equally, particularly in developing countries (15–17). In China, although the Government has attached great importance to green space and has introduced a series of greening policies to guide sustainable urban development (18), how these will affect the dynamic changes in UGS accessibility remains to be determined. Furthermore, as the planning of public service facilities is directly related to social justice and equal distribution of resources, increasing concern about the spatial equality of UGSs has emerged in developing countries, including in China. Therefore, a comprehensive analysis of UGS accessibility that considers changes in the population and transportation network and a discussion of the socioeconomic process underlying the distribution pattern of UGSs can provide suggestions that ensure more reasonable accessibility in urban planning management.

The greening of cities is an important part of the Construction of Ecological Civilization advocated by the Chinese Government. Additionally, “The Healthy China Action (2019–2030)” report issued by the Healthy China Action Promotion Committee proposes requirements for mass sports in China, which in turn, require UGSs to provide a “healthy public space” service function. “Building a healthy China” has become a mainstream and long-term topic at the national strategic level. As an important venue for sports activities, convenient accessibility and fair access are the fundamental principles for UGS spatial allocation. However, the extant research on the changes in UGS distribution is inadequate, particularly the analysis of whether urban inhabitants have equal access to UGSs. Uneven use of UGSs hinders the suitability and effectiveness of green spaces system planning and affects the sustainable development of the whole city. Additionally, although the importance of the role of UGSs in public health is gradually increasing in most east Asian countries that are rapidly aging (19, 20), most studies on the health benefits of UGSs have been conducted in Euro-American countries (19, 20). Previous research on UGS accessibility for people with different socioeconomic statuses (SES) is insufficient in developing countries like China. Furthermore, the effect of different modes of transportation on UGS accessibility has not been adequately addressed. Therefore, this study provides new insights into the dynamic relationship between accessibility and social deprivation and advances the understanding of unequal UGS provision in developing countries.

## Literature Review

### Measurements of Accessibility

In this study, accessibility refers to the convenience of travel from an origin to a specified destination (21). UGS accessibility refers to the degree of difficulty faced by inhabitants in overcoming spatial resistance to reach the green space, indicating the proximity between the inhabitants' place of residence and the publicly accessible urban green spaces (22, 23). The reason for applying the concept of accessibility to the evaluation of UGSs is that traditional indicators such as per capita ratio that have commonly been used in the past do not take into account the convenience offered by the services of parks or green spaces to inhabitants (23). However, accessibility is a people-oriented concept that makes up for this deficiency. In recent years, the research on measuring UGS accessibility has received increasing attention (24, 25). Traditional studies mainly measure the road network distance from residential areas to the nearest facilities (24) to reflect or examine accessibility using different means of transportation (26). However, these studies seldom take into account the factors that combine the supply and demand sides. Accessibility depends on the transportation network and travel mode and includes the supply scale of green space, the demand competition of inhabitants, and other factors, which means that there are more methods available for measuring accessibility.

The commonly used methods can be roughly divided into two categories. The first method is based on spatial interaction, mainly represented by the gravity model, and assumes that the spatial interaction decreases with the spatial interval (travel distance or time) between the destination and origin (17). Spatial interaction increases with the increasing demands of the origin or supply capacity and attractivity of the destination (27). The current research is mainly based on spatial interaction to calculate inhabitants' willingness to visit UGSs (28), but this ignores the impacts on the residential population; that is, the potential population burden of UGSs. The second method is based on cumulative opportunity, which is represented by the two-step floating catchment area method [2SFCA (29) and its extension (30, 31)]. The method search for all inhabitants and public service facilities falls within the catchment area successively, ultimately determining the sum of the supply and demand ratio. Compared with the method of spatial interaction, the method based on cumulative opportunity is more practical for the following two reasons. First, the spatial interaction model may exaggerate the accessibility score of areas with poor accessibility. This results in regional differences being ignored and false identification of underserved areas by policymakers. Additionally, the spatial interaction model is determined by analyzing the distance decay effects of specific travel modes, which are difficult to obtain. Although the analysis results of the two-step mobile search method are relatively objective, the extension of the 2SFCA, which is based on multiple travel modes, is seldom applied in the research on green space accessibility. The issue of spatial equality of UGSs has been addressed by a series of studies that have examined traveling to UGSs using a pre-set single (or uniform) transport mode (28, 31, 32) or within a specific distance threshold (29, 31). Other scholars have noted that accessibility should be highly sensitive to different transport modes (33). To date, however, there are limited explanations for the mechanisms involved in the dynamic changes in accessibility by multi-mode transport over time.

### Deprivation of Urban Green Spaces

Deprivation can be described by indicators of community socioeconomic disadvantages (e.g., earning power, education, political rights, housing type, and population structure). UGSs are often not distributed equitably, and the unfair spatial distribution is closely related to social injustice (34). A growing body of literature has demonstrated that the spatial distribution of UGSs has been stratified for population segments of different ages, genders, ethnic backgrounds, social statuses, incomes, and other axes. They contend that accessibility varies according to SES. Scholars have also evaluated spatial equality by measuring accessibility (35) and considering the differences in social groups (17). The accessibility heterogeneity between different socioeconomic groups usually reflects social equality, converting the research focus of UGS accessibility from “spatial equality” to “social equality.” The majority of empirical research on UGSs has demonstrated the characteristics of the inverse care law, where urban public resource allocation centers on high-income residents. For example, UGSs are characterized by being concentrated in high-income neighborhoods (36–38) and being available to people who own houses (39), mainstream ethnic groups (24, 40), and people of high socioeconomic status (41, 42). This demonstrates a corresponding relationship between the injustice caused by the spatial distribution of UGSs and the social injustice of deprivation, while the inverse care law reflected in the spatial distribution of UGSs indicates deprivation. However, some studies have demonstrated that the correlation between UGS accessibility and SES is insignificant (35, 43, 44), while others have concluded otherwise (45, 46).

In conclusion, the above variance in results is not limited to the different social backgrounds or different temporal intervals of the previous studies. They are also attributable to technical limitations such as the inability of regional units to represent the service areas of the UGSs, aggregation errors caused by the Modifiable Areal Unit Problem (MAUP; the measurement of spatial distributions according to different geographical divisions of a given number of areas induces different results), and limitations caused by distance measurement options (47). Furthermore, for accessibility estimation, if the different transportation modes of inhabitants are neglected, the outcomes are one-sided and unrealistic, which causes misidentification of the deprivation of UGSs for disadvantaged groups. The uncertainty of the research results weakens the evidence base for effective policy interventions to eliminate the deprivation and results in less effective mitigation programs.

The few empirical studies that have focused on community deprivation of urban green spaces in China have mainly been conducted in metropolitan cities like Beijing, Shanghai, and Shenzhen. However, most of these megacity or megacity behemoths have implemented stricter policies for the control of migrants, and the permanent population growth has slowed down, and in some cases, it is negative. With the total amount of green space unchanged, the per capita possession could increase. On the contrary, as the residency restrictions on migrants have been relaxed in provincial capitals and other cities, the process of rapid population agglomeration toward some mid-sized cities is still ongoing. The local governments of most cities also intend to strengthen the construction of urban green space. Under the background of rapid urbanization, whether the behavior of the city government relieves the deprivation of green space needs to be clarified by relevant research. Additionally, while plains dominate the topography of the case city, other complex topography may restrict the space for urban development, and the area of land available for green space construction might be limited.

Finally, the previous studies have adopted relatively static perspectives, and there is no rigorous explanation for selecting time nodes. Nevertheless, it is critical for dynamic research to choose more representative time nodes to test the effectiveness of the local Government's green space planning implementation. In summary, in the research on dynamic change in UGS, we need to shift our focus to non-megacities with complex topography. Meanwhile, we must be particularly cautious in choosing the research time nodes so that the research results can truly reflect the changes in spatial and social equity of UGSs during the process of green space planning.

### The Present Study

In this study, the central urban area of Fuzhou (119°E, 26°N) was selected as a representative case with longer and more meaningful research temporal intervals, as this city is set in a valley surrounded by rolling hills, and the available space for urban expansion is limited. Fuzhou was deemed a National Ecological Civilization Pilot Zone, with the highest green space coverage rate nationwide. Fuzhou has been awarded titles such as National Garden City, National Model City for Afforestation, National Ecological City, and National Forest City. Fuzhou's long history and geographical conditions have laid the foundation for the establishment of UGSs. Meanwhile, the local Government also attaches great importance to green space construction. According to the specific goals for constructing green space proposed by the green spaces system planning of Fuzhou city (2013–2020), the per capita green area for the central urban area was 15 m^2^. While Fuzhou's UGS surface per capita is higher than most other cities in China, equitable access to UGSs cannot be captured using the total UGS surface per capita (48).

Additionally, over the past few years, the city has witnessed burgeoning population growth, which has accelerated urban land-use conversion and infrastructure development. Therefore, whether the excellent natural base combined with previous efforts results in an equal supply of green space or requires further adjustments is uncertain and needs to be explored in depth. To provide useful suggestions for urban planning by exploring UGS accessibility in southeast China and its dynamic relationship with social and economic factors, fine-scale demographic datasets at the community level were collected from 2012 to 2020. This timeframe corresponds to Fuzhou city's green spaces system planning (2013–2020) undertaken by the local Government. It is meaningful to analyze the dynamic changes in green space accessibility from the perspective of social equality to lay the foundations for future urban development.

**Figure 1:**
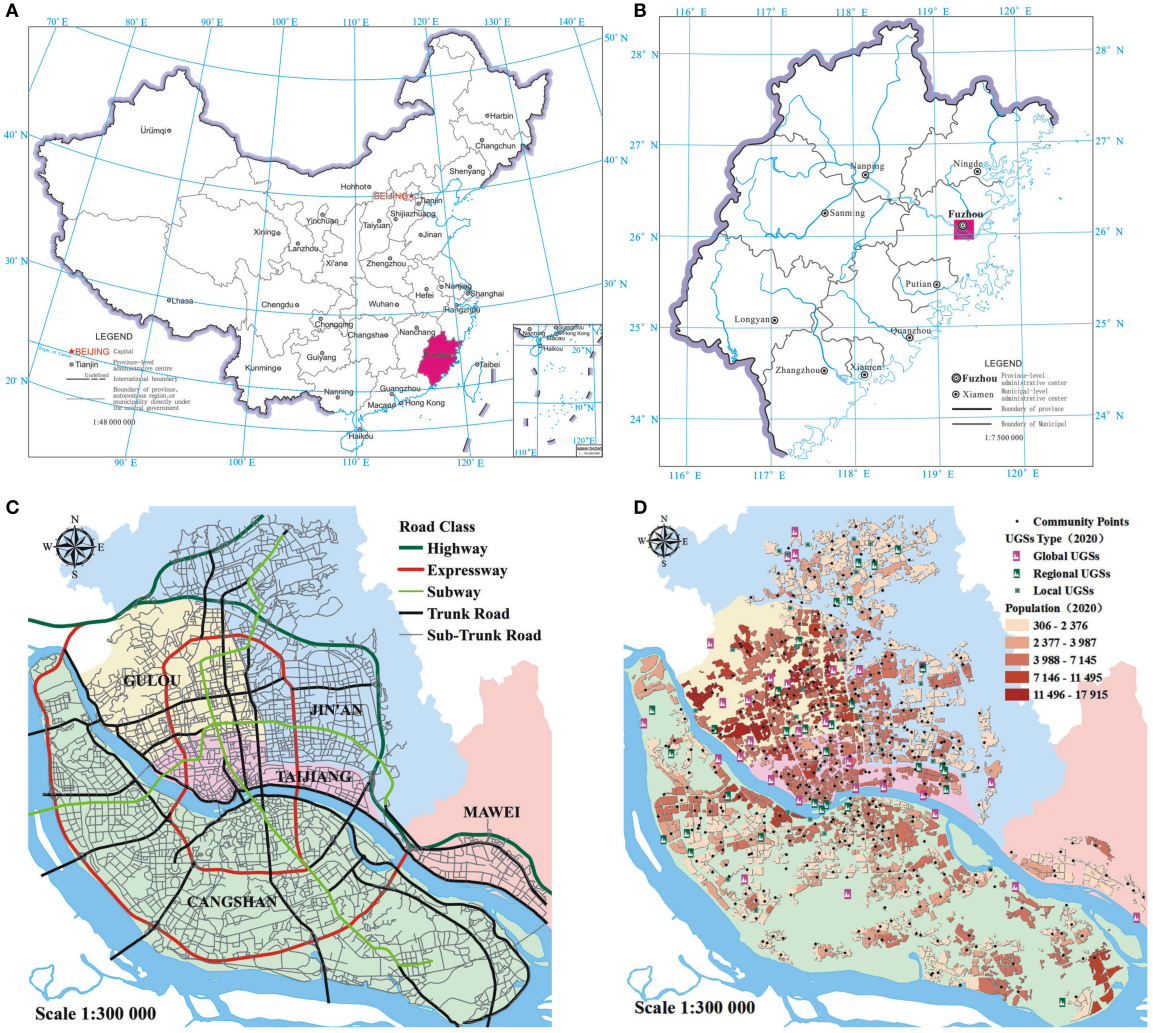
Location of the study area in China and the UGSs, population demands, and transportation systems within it. The collection and division of green spaces for specific years.

As illustrated in Figure 1, Fuzhou is the provincial capital of Fujian province, which is located in China's southeast coastal area, faces Taiwan, and has beautiful landscapes and a pleasant ecological environment. The central urban area of Fuzhou consists of five districts with high transport network density and several UGSs belonging to different categories (Figure 1). From 2012 to 2020, the city experienced rapid urbanization, population growth, and infrastructure development. For example, two metro lines were built and implemented successively, and the average transport network density grew by approximately 50% from 2012 to 2020. Based on Fuzhou City's (2011–2020) urban planning, the urban population of Fuzhou central city was expected to be 4.1 million at the end of 2020, increasing by over a million compared with the population in 2012. Although the green space continues to be planned and constructed, there is still no empirical research on whether the increase can keep pace with the increase in the inhabitants' demand and guarantee equal well-being to all inhabitants. Therefore, the specific objectives of this study are as follows: (1) analyze the accessibility to UGSs by multi-mode transport over time; (2) reveal the social inequalities by exploring the dynamic relationship between community deprivation and transport modes in relation to varying accessibility of UGSs; and (3) enrich the current literature on geographic accessibility and provide spatial insights into deprivation to formulate more contextualized and effective plans for policymakers.

## Data and Methods

### Data and Pre-processing

UGS spatial data were obtained through remote sensing (RS) image interpretation. Using remote sensing image interpretation, combined with Map World-Fujian (the provincial level branch of the national platform for common geospatial information system) in related years and green space system planning, the quantity and location of UGSs were verified and validated through a field investigation. The complete data are listed in Table 1.

**Table 1:** Data type, data content, and its main application in the study.

**Data type**	**Data content and source**	**Main application**
Remote sensing imagery	Imagery provided by Resources satellite three (ZY-3), acquisition date = March 9, 2013 at a resolution of 0.5 m; Gaofen-2 satellite (GF-2), acquisition date = December 7, 2016 at a resolution of 0.8 m; Gaofen-2 satellite (GF-2) acquisition date = February 2, 2020 at a resolution of 0.8 m	For the collection of UGSs and verification of road network in 2012, 2016, and 2020
Spatial data and attribute data for the UGSs	The data of UGSs origin from Map World-Fujian and the green space system planning of Fuzhou city	For the acquisition of UGSs combined with imagery; As the basis of location, area, and classification for UGSs
Travel survey that assesses purpose of travel to UGSs	Field investigation and questionnaire	To estimate the threshold travel time by multi-mode transport; To check the actual situation of UGSs
Road network and community location	Fujian fundamental geographic information database	For the acquisition and vectorization of road networks and community area in 2012, 2016, and 2020
Demographic properties	The data of population characteristics obtained from the population information management system	As the relevant attribute information for the community

**Figure 2:**
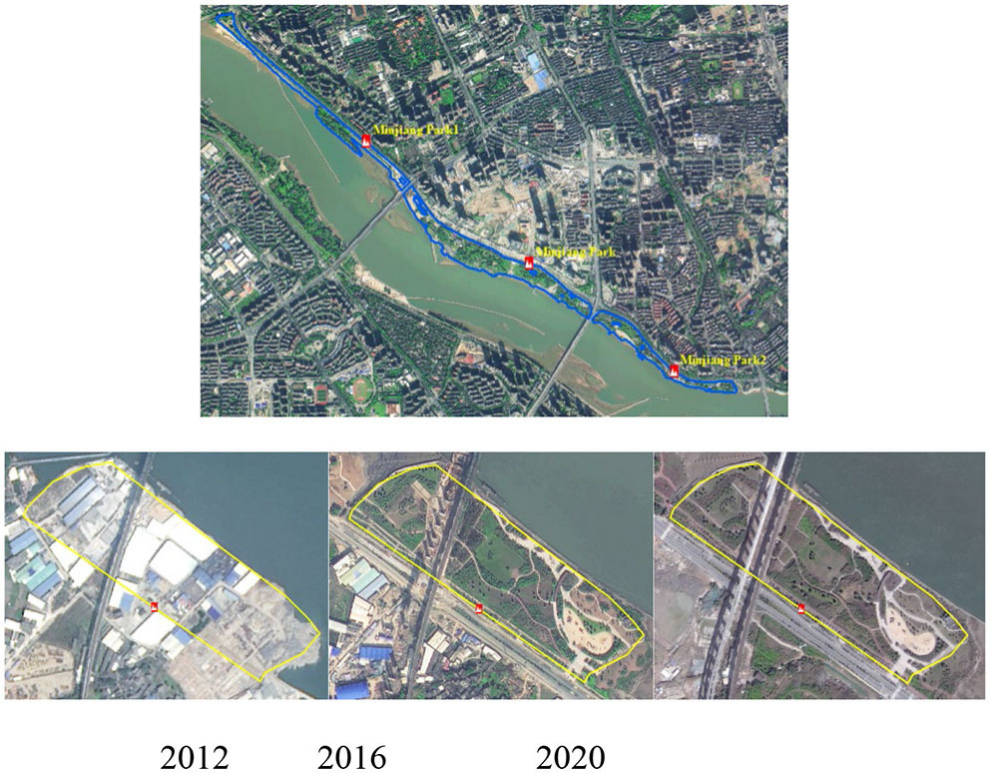
The collection and division of green spaces for specific years.

The base year (2012) of the relevant attribute data, such as the area and classifications of UGSs, were mainly obtained through an actuality investigation of Fuzhou city's green space system planning (2013–2020). Other extensions and newly developed UGSs in 2016 and 2020 were jointly determined from remote sensing (RS) interpretation, together with the green space system planning. The classifications of green spaces that were not included in the planning were mainly determined based on their area. Additionally, considering the rationality of the accessibility analysis results, belt-like open green spaces were divided into several parts according to their multi-entrances. The details of the data collection are illustrated in Figure 2, demonstrating that the number of UGSs has been increasing annually and had reached 90 by 2020.

Furthermore, road availability for multi-mode transport was determined by RS imagery, traffic rules, and field investigations. The pre-processing for road networks included the examination and verification of road directions and built or under-construction roads. Road network access and topological relationships were evaluated depending on the actual situation. Roads were divided into different types, such as highways, city circles, and auxiliary roads. According to different travel modes, the speed was set for different road categories according to the corresponding national standards and local reality. As illustrated in Table 2, a portion of the values was NULL, meaning that specific roads could not be accessed by the related travel modes. A dataset on the demographic properties at the community level in 2012, 2016, and 2020 was obtained for the deprivation analysis. The principles of objectivity, representativeness, and briefness were then taken into adequate consideration for selecting the indicators. Finally, variables representing four categories (age, education, work experience, and household registration status at the community level) were selected from a series of potential indicators (Table 3). These methods serve to make the conclusions more convincing.

**Table 2:** Travel costs of different road classifications by multi-mode transport.

**Road classification**	**Highway**	**Expressway**	**Trunk road**			**Sub-trunk road**		**Subway**	
	**Highway**	**City circle**	**Auxiliary road**	**Main Street**	**Minor street**	**Ramp**	**Feeder**	**Internal road**	**Subway**
Public transport	50	40	30	30	30	20	20	20	50
Cycling	-	-	-	15	15	-	15	15	-
Walking	-	-	-	5	5	-	5	5	-

**Table 3:** Descriptive statistics of the selected socioeconomic variables of the community.

**Variables**	**Explanation**	**Max**	**Min**	**SD**	**Mean**
Older population	Proportion of people aged over 65 years	0.44	0.04	0.07	0.24
Unemployment	Proportion of unemployed people	0.88	0.01	0.15	0.19
Illiteracy	Proportion of illiterate people	0.34	0.04	0.06	0.15
Less educated population	Proportion of people with degree lower than junior middle school	0.95	0.10	0.15	0.46
Floating population	Proportion of floating people	0.89	0	0.20	0.24

### Multi-Mode 2SFCA for Spatial Accessibility to the UGSs

The study proposes a multiple transportation mode method called the multi-mode 2SFCA, which is based on the framework of the 2SFCA. The multi-mode method is implemented using the following two steps:

Step 1: For each UGS *j*, search for all populations that fall within different predefined threshold travel times by mode from j, and on that basis, draw different catchment areas. Then, compute the supply-demand ratio *V*_*j*_ within the catchment areas

(1) $$\begin{array}{r} V_{j}=\frac{S_{j}}{\sum_{k\in d_{jk}\left(M_{1}\right)\leq d_{0}\left(M_{1}\right)}P_{k{,M}_{1}}+\sum_{k\in d_{jk}\left(M_{2}\right)\leq d_{0}\left(M_{2}\right)}P_{k{,M}_{1}}+\sum_{k\in d_{jk}\left(M_{3}\right)\leq d_{0}\left(M_{3}\right)}P_{k{,M}_{3}}} \end{array}$$

where *d*_*jk*_ (*M*_*n*_) is the travel time by mode *M*_*n*_ between UGS *j* and community *k*; and *d*_0_(*M*_*n*_) is a predefined threshold travel time from *j* by mode *M*_n_. In this way, several catchment areas by mode can be drawn around UGS *j*. *V*_*j*_ reflects the supply availability of UGS *j* that can be offered to a person who can reach it by different transportation modes.

Step 2: For each population community *i*, search for all UGSs (*j*) that fall within the threshold travel time by mode from *i*. Thus, draw the different catchment areas again. The supply-demand ratios within different catchments are weighted by community population size, and the weighted values are added together to calculate the overall accessibility (*A*_*i*_) of community *i*.

Thus, the accessibility of a community is dependent on the distribution of UGSs, as well as on its population, road network characteristics, and uneven public transport supply:

(2) $$\begin{array}{r} A_{i}=\frac{P_{i{,M}_{1}}\sum_{j\in d_{ij}\left(M_{1}\right)\leq d_{0}\left(M_{1}\right)}V_{j}+P_{i{,M}_{2}}\sum_{j\in d_{ij}\left(M_{2}\right)\leq d_{0}\left(M_{2}\right)}V_{j}+P_{i{,M}_{3}}\sum_{j\in d_{ij}\left(M_{3}\right)\leq d_{0}\left(M_{3}\right)}V_{j}}{\sum_{v=1}^{3}P_{i,M_{v}}} \end{array}$$

According to the Traffic Analysis Reports for Major Cities in China proposed by AutoNavi and the state information center, Fuzhou has a high green travel willingness index. The cycling travel willingness index in Fuzhou ranks third in China, and the demand for public transport, such as buses and subways, is quite large.

The existing reviews revealed significant spatial heterogeneity in UGS accessibility via walking and public transit. They also confirmed that significant social inequalities widely exist except for the private car mode (17). Furthermore, given the difficulty of obtaining data on the number of cars per family, the study integrates walking, cycling, and public transportation as a multi-mode choice of transport to study UGS accessibility. The model aims to stimulate the status of UGS accessibility. To minimize the bias introduced by MAUP, the multi-temporal analysis was conducted using the minimum possible scale in the given conditions.

**Figure 3:**
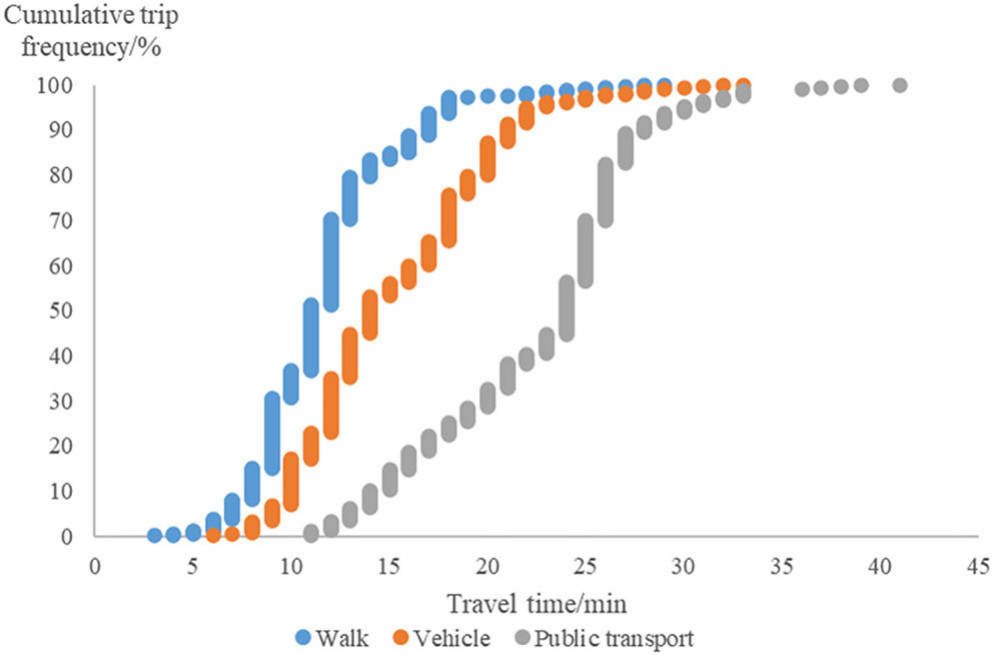
The cumulative trip frequency of UGSs-oriented trips against the travel time by multi-mode transport.

During the analysis, it was apparent that selecting a reasonable threshold travel time is the key point for using the multi-mode 2SFCA. To estimate the threshold travel time (by mode) empirically, we used a questionnaire. The results demonstrated that the travel time to UGSs varied according to the travel mode. We could then derive the frequency distribution of trips against travel time (Figure 3). It was found that the frequency of trips decreased as travel time increased, and the inflection points for walking, cycling, and public transport were 18, 23, and 33 min, respectively. The 18-min travel time approximates to the cut-off walking trip cost to UGSs that has been used in several previous studies (32, 34). Additionally, the 33-min public transport trip covered approximately 97% of city-level UGSs in the study area, which prevented some sub-districts from having zero values. Therefore, we set the threshold travel time at 18, 23, and 33 min for walking, cycling, and public transport, respectively.

### KRLS for Dynamic Changes in Deprivation Associated With UGS Accessibility

A challenge for analyzing dynamic changes in deprivation is that the marginal effects are always heterogeneous across the covariate space that cannot be appropriately handled by generalized linear models (GLMs). Furthermore, GLMs have significant shortcomings as the existence of highly interdependent relationships among the demographic properties of the community does not conform to the inherent assumptions (49). While more flexible methods that do not rely on linear or additive hypotheses have occasionally been proposed, none of these have received widespread acceptance due to their lack of applicability and interpretability (50, 51). Therefore, a growing body of literature addresses social issues using machine learning algorithms, which provide a strong capability to deal with non-linear and interactive data without strict theoretical and parametric assumptions (52, 53). In addition to the advantages mentioned above, Kernel regularized least squares (KRLS) regression provides closed-form estimates for the pointwise partial derivatives that characterize the marginal effects of each independent variable across the covariate space. The method also allows for interpretation and inference in ways similar to that of GLMs (50). Given the above, the study utilizes the KRLS to analyze the dynamic relationship between community deprivation and UGS accessibility. The representative variables were selected from a set of potential variables about SES, and they were divided into several categories. The best subset selection was then obtained by going through all the permutations.

## Results

### The Interpretation of Transport Modes—Varying Accessibility of UGSs Over Time

**Figure 4:**
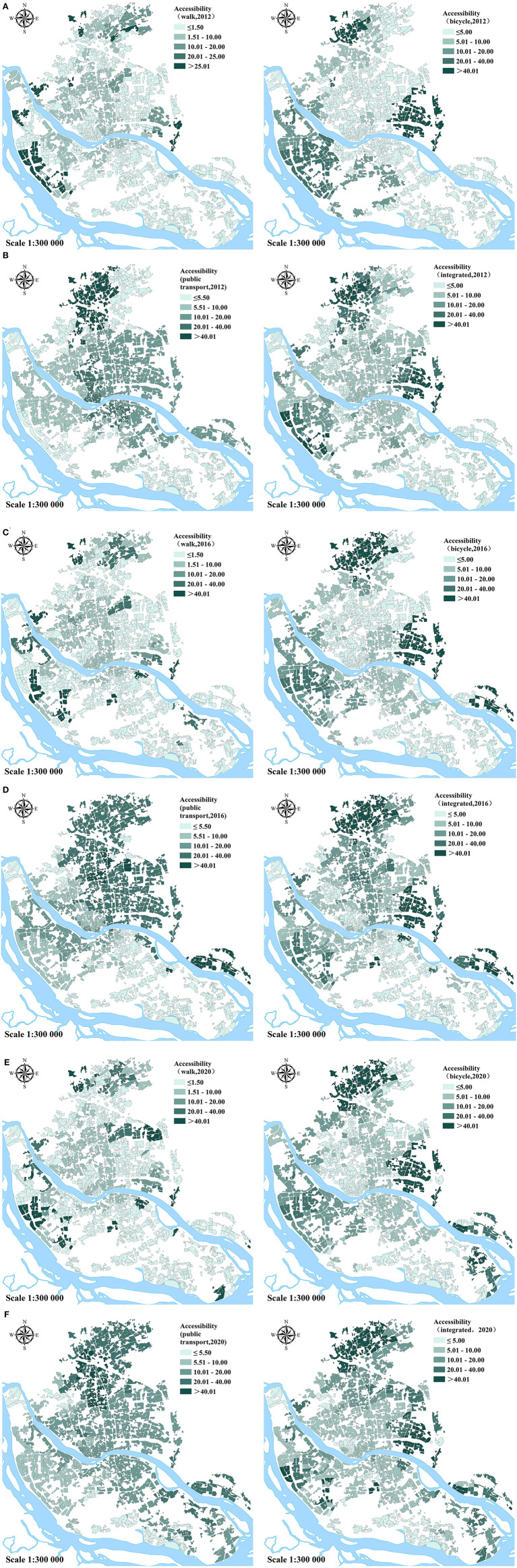
Spatial heterogeneity of varying accessibility of UGSs based on transport modes over time: by the walking, cycling, public transport, and integrated modes in 2012, 2016, and 2020, respectively (unit: square meters per person).

The central urban area of Fuzhou city has witnessed an overall improvement trend in UGS accessibility from 2012 to 2020 using multi-mode transport (Figure 4). The spatial heterogeneity for the UGS accessibility of the specified year indicates great variations among different transport modes. The regions with high accessibility by walking mode are scattered around the urban area. In the cycling mode, the high-accessibility regions are in the western, eastern, and northern periphery areas, and these surround the low-accessibility region in the core urban areas, forming a semi-enclosed spatial pattern. The difference between the high-value regions and their surrounding regions is more pronounced in the north and east, where the major UGSs are located. However, the distribution of high accessibility by public transit mode has a core urban spatial orientation, the complete opposite of the above two modes. The high accessibility by public transit appears as contiguous spatial units in the core urban areas, and the advantages in the accessibility of the periphery by walking mode and cycling mode weaken. The spatial heterogeneity of the integrated travel modes is mostly similar to the cycling mode pattern through which access is limited in the core urban areas. The spatial pattern of UGS accessibility also varies over time by the same mode, and the overall dynamic process leads to a decrease in the area with limited accessibility. For example, the high accessibility by public transport is concentrated around the core urban areas and then expands, particularly to the southeast. The semi-enclosed spatial pattern for cycling tends to weaken gradually, for which the accessibility of the core urban areas continuously improves. Thus, UGS accessibility becomes evenly distributed over time.

A previous study identified the relative role of local contributors, such as population and transport networks, on accessibility changes (29). The present study further measured the dynamic changes in UGS accessibility in response to the evolution of the UGS classification system by multi-mode transport, which has rarely been investigated. As illustrated in Table 4, although the accessibility of city-level UGSs by cycling or public transit has declined slightly from 2016 to 2020, which undoubtedly has an adverse effect on the total growth, accessibility has grown overall and for most UGS classifications during the time intervals. While the accessibility for the community-level and district-level UGSs has achieved steady growth, the inhabitants have increasingly easy accessibility to all UGSs classifications by walking.

**Table 4:** The dynamic changes in the accessibility for different classifications of UGSs during the study period.

	**2012**				**2016**				**2020**			
	**Total**	**Level-1**	**Level-2**	**Level-3**	**Total**	**Level-1**	**Level-2**	**Level-3**	**Total**	**Level-1**	**Level-2**	**Level-3**
Walking	11.59	0.22	2.00	9.36	12.17	0.26	1.88	10.03	13.13	0.40	2.31	10.43
Cycling	17.99	0.22	2.00	15.76	18.78	0.26	2.23	16.29	18.68	0.40	2.31	15.97
Public transport	17.99	0.22	2.00	15.76	18.78	0.26	2.23	16.29	18.68	0.40	2.31	15.97
Integrated	15.86	0.22	2.00	13.63	16.58	0.26	2.11	14.21	16.83	0.40	2.31	14.12

*level-1, level-2, level-3 represent the UGSs of community-level, district-level, and city-level individually*.

**Figure 5:**
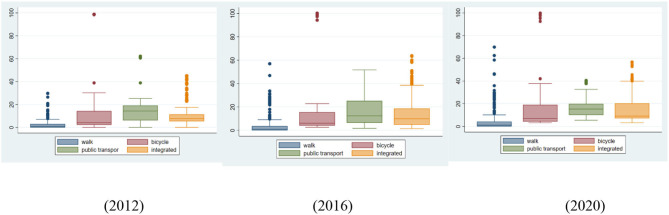
Box plots for the dynamic changes in the accessibility of UGSs by multi-mode transport.

The detailed dynamic changes in UGS accessibility among communities are presented in Figure 5 for single (walking, cycling, and public transport) and integrated travel modes. The UGS accessibility data distribution varies greatly with multi-mode transport. Apart from the more concentrated data for walking, the extreme values account for a higher proportion than for other travel modes. During the study period, the data distribution by public transport changed dramatically. Within the boxes, the upper 50% of values by public transport occupied a larger proportion from 2012 to 2016 and then narrowed to approximately equal from 2016 to 2020. Furthermore, by the public transport mode, the box and range of the upper quartile stretch and compress. These processes can be described as a global surge following local enhancement.

### The Correlation Between UGS Accessibility and Community Deprivation Varies With Multi-Travel Modes Over Time

Tables 5–7 illustrate the relationship between community deprivation and UGS accessibility in 2012, 2016, and 2020. The average column indicates the averages of the marginal effect over the covariate space on the change in UGS accessibility. It cannot reflect the heterogeneous effects of covariates across the units and levels of other covariates for which the relationship between the structural characteristics and UGS accessibility changes in community deprivation had been confirmed. As illustrated in the last three columns, the marginal effects vary by quantile and are listed using P25, P50, and P75. For example, the correlation between less educated people and UGS accessibility changes by walking is always negative. However, the heterogeneity in the pointwise marginal effects is apparent: the average marginal effect in the fourth row of Table 5 under the average column is −0.09; in the first quartile, a one-unit increase in the proportion of less-educated people is associated with a 0.17 percentage point decrease in UGS accessibility, while in the third quartile, it is associated with only a 0.03 percentage point decrease in UGS accessibility. The median of the marginal effects is −0.10.

**Table 5:** Correlation between community deprivation and UGS accessibility by multi-mode transport in 2012.

	**Walking**				**Cycling**				**Public transport**				**Integrated**			
	**Avg**.	**P25**	**P50**	**P75**	**Avg**.	**P25**	**P50**	**P75**	**Avg**.	**P25**	**P50**	**P75**	**Avg**.	**P25**	**P50**	**P75**
Aged >60	0.06	−0.03	0.04	0.11	0.08	−0.20	−0.08	0.17	0.21^***^	0.02	0.21	0.36	0.20^***^	0.06	0.15	0.29
Unemployed	−0.06	−0.14	−0.06	0.04	−0.07	−0.26	−0.08	0.06	0.21^***^	−0.06	0.13	0.49	0.06	−0.07	0.03	0.20
Illiterate	−0.08	−0.16	−0.07	−0.01	0.05	−0.06	0.04	0.19	−0.22^***^	−0.41	−0.26	−0.06	0.02	−0.15	−0.02	0.20
Less educated	−0.09^*^	−0.17	−0.10	−0.03	−0.25^*^	−0.36	−0.06	0.06	−0.14^**^	−0.32	−0.05	0.08	−0.29^***^	−0.50	−0.29	−0.02
Migrants	−0.05	−0.14	−0.06	0.03	−0.06	−0.14	−0.03	0.07	−0.05	−0.20	−0.04	0.12	−0.06	−0.15	−0.02	0.08

* *p < 0.05*,

** *p < 0.01*,

*** * p < 0.001*.

**Table 6:** Correlation between community deprivation and UGS accessibility by multi-mode transport in 2016.

	**Walking**				**Cycling**				**Public transport**				**Integrated**			
	**Avg**.	**P25**	**P50**	**P75**	**Avg**.	**P25**	**P50**	**P75**	**Avg**.	**P25**	**P50**	**P75**	**Avg**.	**P25**	**P50**	**P75**
Aged >60	0.15^*^	0.08	0.13	0.17	0.02	−0.02	0.01	0.06	−0.11^*^	−0.23	−0.10	0.01	−0.09	−0.16	−0.07	−0.01
Unemployed	−0.04	−0.06	−0.03	−0.01	0.08^*^	0.02	0.07	0.14	0.08	−0.06	0.06	0.19	0.08	0.01	0.09	0.16
illiterate	0.03	−0.01	0.02	0.09	0.08^*^	0.04	0.09	0.12	0.23^***^	0.07	0.20	0.44	0.09	−0.01	0.09	0.17
Less educated	0.01	−0.05	−0.01	0.04	0.06	−0.02	0.05	0.13	−0.06	−0.15	−0.03	0.07	−0.09	−0.17	−0.10	−0.02
Migrants	0.04	0.01	0.06	0.11	0.03	−0.01	0.04	0.07	0.14^**^	0.01	0.13	0.27	0.09^*^	−0.01	0.07	0.16

* *p < 0.05*,

** *p < 0.01*,

*** *p < 0.001*.

**Table 7:** Correlation between community deprivation and UGS accessibility by multi-mode transport in 2020.

	**Walking**				**Cycling**				**Public transport**				**Integrated**			
	**Avg**.	**P25**	**P50**	**P75**	**Avg**.	**P25**	**P50**	**P75**	**Avg**.	**P25**	**P50**	**P75**	**Avg**.	**P25**	**P50**	**P75**
Aged >60	0.11^*^	−0.01	0.08	0.22	−0.07	−0.25	−0.07	0.11	−0.09	−0.11	0.08	0.19	−0.13^*^	−0.27	−0.07	0.04
Unemployed	0.01	−0.08	0.01	0.12	−0.15^**^	−0.34	−0.16	0.04	−0.13	−0.35	−0.11	0.10	−0.23^***^	−0.50	−0.21	0.01
Illiteracy	−0.05	−0.22	−0.08	0.15	−0.20^***^	−0.39	−0.12	0.06	−0.05	−0.33	−0.05	0.11	−0.28^***^	−0.54	−0.14	0.02
Less educated	−0.03	−0.24	−0.03	0.18	−0.24^***^	−0.66	−0.06	0.19	−0.10	−0.48	−0.10	0.19	−0.27^***^	−0.53	−0.22	−0.07
Migrants	−0.14^*^	−0.39	−0.12	0.03	−0.08	−0.25	−0.02	0.17	0.10	−0.12	0.08	0.27	−0.01	−0.17	0.04	0.19

* *p < 0.05*,

** *p < 0.01*,

*** *p < 0.001*.

Table 5 demonstrates that community deprivation was evident for some disadvantaged groups in 2012. For example, educationally restricted people (including illiterate and less educated people) negatively correlate with UGS accessibility by multi-mode transport, most notably for less educated people. This indicates that communities with a lower proportion of people with more than nine years of education always have restricted access to UGSs in most travel modes.

As revealed in Tables 5–7, the comparative advantages of accessibility for older people seem to gradually weaken or even have disadvantages, except for the walking mode. However, with the aging trend in most communities, the issue mentioned above is more serious than what was observed. Educationally restricted communities are moving toward better outcomes in 2016 compared with 2012. However, the accessibility of communities with more aged people is deteriorating, particularly by public transport. In 2020, more disadvantaged groups, including unemployed, illiterate, less educated, and migrant community members, presented negative correlations with UGS accessibility, even though some were non-significant.

## Discussion and Conclusions

### Understanding the Existing and Improving Spatial Inequality of UGS Accessibility That Varies With Multi-Travel Modes Over Time

Overall, UGS accessibility continues to improve, while the region with restricted accessibility has gradually decreased. Fuzhou inhabitants have more opportunities to visit community-level and district-level UGSs, regardless of their chosen travel modes. The spatial distribution of UGSs is optimized through the expansion and spatial rearrangement of green spaces. In particular, inhabitants of the southeastern region, which is also along the city's development corridor, have easier access to UGSs due to the newly built green space in this area. Although the central urban area population has rapidly increased, the increased traffic network density and layout optimization of UGSs allow convenient travel to UGSs. As large-scale UGSs are primarily distributed in the urban periphery, the core urban areas have relatively poor accessibility by walking or cycling. However, core urban areas are always intensive bus route zones, making it very convenient for people to reach UGSs by public transport, particularly with the continuous expansion of Fuzhou subway lines.

The Healthy China Action (2019–2030) report advocates a healthy lifestyle, insisting on low-carbon travel; prioritizing walking, cycling, or public transportation; and advocating more shared transportation. However, the multi-mode practice continues to lag in UGS accessibility research. In this study, the catchment area for walking is approximately 15 min on the physical exercise scale mentioned in the action program. Nonetheless, the results presented in Figure 1 illustrate that accessibility by walking is generally inadequate. The spatial heterogeneity by cycling mode is more significant, indicating serious spatial inequality. By public transport mode, the region with the best access to UGSs tends to be consistent with the region with high levels of public transport density, which may be unfavorable to disadvantaged groups who live on the urban periphery where the public transport system needs to be further developed. Given that the spatial patterns of UGS accessibility vary with multi-mode transport, the promotion of spatial equality should not be confined to the optimization of allocation of UGSs.

Additionally, to date, more than half of the communities continue to have a limited per capita area of UGSs within the catchment, which is less than the required area for a National Ecological Garden City (beyond 12 per capita area). Confined by the pressure of rapid population growth, UGS accessibility tended to be stable in the last 4 years (2016–2020) after the definite increase, which was mainly concentrated in the previous 4 years (2012–2016). Due to the large scale of the green spaces distributed in the periphery of the urban areas, the ideal accessibility to city-level UGSs depends on cycling or public transport. In contrast, as community-level UGSs are mostly located in the core urban areas with high population densities, the accessibility and potential to improve are limited.

### Interpretation of Social Equality From the Perspective of UGS Accessibility

The limited access to UGSs for disadvantaged groups at the community level reflects the existence of community deprivation. The specific disadvantaged groups have different accessibility by multi-mode transport, and this access also changes with time. Overall, significant deprivation mainly exists for less educated people or those using the cycling and integrated travel modes. Less-educated people tend to live in communities characterized by lower coverage of green spaces and fragmented and narrow lanes (54). This explains the community deprivation in UGS accessibility in the cycling travel mode, which further affects deprivation in the integrated travel mode.

The dynamic changes in accessibility cannot be captured completely if only two time nodes are selected for comparison (the origin and destination). However, even with three or more time nodes, the results could be insufficiently convincing if the time intervals are short. Therefore, this study selected three representative time nodes for which the time interval was sufficiently long to observe changes. Tables 8, 9 illustrate the relationships between community deprivation changes and dynamic changes in UGS accessibility between the last 4 years (2016–2020) and the previous 4 years (2012–2016). For a community with a growing proportion of disadvantaged groups, such as unemployed people and migrants, UGS accessibility by the most modes of transport continues to grow from 2012 to 2016, even though not all modes were significant. However, there is scarcely any change in equity from 2016 to 2020 except for the accessibility by walking mode for unemployed people, but 2016 appears to have been an important turning point in community deprivation of UGSs. For example, the dynamic correlation between illiteracy and UGS accessibility showed more serious significant negative before and after 2016, which means that changes in the accessibility dynamic failed to keep up with the changes in illiteracy. Combined with the accessibility changes in relation to community deprivation in 2012, 2016, and 2020, the correlation coefficient changed from partially positive to totally positive and then totally negative, indicating that UGS accessibility may not be sufficient to make up for the inequality of illiteracy. The access to UGSs among inhabitants with different SES is relatively equitable in 2016.

**Table 8:** Correlation between community deprivation changes and the dynamic changes in the UGS accessibility from 2012 to 2016.

	**Walking**				**Cycling**				**Public transport**				**Integrated**			
	**Avg**.	**P25**	**P50**	**P75**	**Avg**.	**P25**	**P50**	**P75**	**Avg**.	**P25**	**P50**	**P75**	**Avg**.	**P25**	**P50**	**P75**
Aged > 60	0.17^***^	0.09	0.18	0.25	0.02	−0.05	0.03	0.09	−0.17^**^	−0.31	−0.16	−0.02	−0.09^*^	−0.14	−0.09	−0.03
Unemployed	−0.07	−0.11	−0.07	−0.03	0.05	−0.09	0.06	0.20	0.06	−0.07	0.05	0.20	0.06	0.01	0.06	0.12
Illiterate	−0.01	−0.05	−0.01	0.04	0.08	0.01	0.10	0.17	−0.07	−0.22	−0.03	0.12	−0.04	−0.10	−0.03	0.03
Less educated	−0.07	−0.14	−0.04	0.01	0.04	−0.05	0.02	0.09	−0.02	−0.18	0.01	0.17	−0.06	−0.13	−0.05	0.01
Migrants	−0.03	−0.09	−0.04	0.02	0.01	−0.05	0.02	0.07	0.22^***^	0.01	0.23	0.48	0.08^*^	0.02	0.10	0.16

* *p < 0.05*,

** *p < 0.01*,

*** *p < 0.001*.

**Table 9:** Correlation between community deprivation changes and the dynamic changes in the UGS accessibility from 2016 to 2020.

	**Walking**				**Cycling**				**Public transport**				**Integrated**			
	**Avg**.	**P25**	**P50**	**P75**	**Avg**.	**P25**	**P50**	**P75**	**Avg**.	**P25**	**P50**	**P75**	**Avg**.	**P25**	**P50**	**P75**
Aged >60	0.04	−0.01	0.06	0.11	−0.04	−0.08	−0.04	0.01	0.02	−0.09	0.04	0.13	0.03	−0.08	0.03	0.15
Unemployed	0.09^*^	0.04	0.08	0.13	−0.05	−0.08	−0.04	−0.01	0.03	−0.10	0.04	0.17	0.02	−0.05	0	0.10
Illiterate	0.03	−0.03	0.02	0.08	−0.02	−0.05	−0.02	0.02	−0.07	−0.19	−0.07	0.06	−0.11^*^	−0.18	−0.11	−0.04
Less educated	−0.05	−0.07	−0.02	0.02	−0.04	−0.07	−0.04	0.01	−0.08	−0.24	−0.08	0.07	−0.08	−0.17	−0.08	0.01
migrants	0.02	−0.04	0.03	0.10	−0.05	−0. 08	−0.06	−0.03	0.06	−0.11	0.05	0.24	0.06	−0.02	0.06	0.15

* *p < 0.05*.

In other words, within the study period, equality of access to UGSs tended to first increase and then decrease. The social inequality from the perspective of UGS accessibility is similar to the green gentrification that has garnered widespread concern, particularly in Western countries (48, 55). The issue is closely related to the marginalization of disadvantaged groups and residential segregation, but it is frequently ignored in China (56). The re-emergence of inequality serves as a reminder to the Government that current green planning should be revised to prevent the realization of the inverse care law. It is noteworthy that the significant negative correlation between disadvantaged groups and UGS accessibility disappeared along with the rapid development of public transport, which could be seen as manifesting the trend toward a more equitable society. It can be said that the opening and operation of Fuzhou Subway Lines increases transportation convenience for disadvantaged groups.

In conclusion, this study revealed the periodic characteristics of community deprivation, which has seldom been discussed to date. Although rare studies on the same topic present different conclusions (20, 29), it has been difficult to provide a broad overview of the development of deprivation and even more difficult to determine when UGS accessibility becomes more favorable for disadvantaged groups. The limitations in the timelines create difficulties for policymakers in terms of formulating effective and timely responses. With the aid of long-time-series data, this study found that the degree of community deprivation is alleviated at the beginning but becomes more serious later. This change demonstrates the importance of studying temporal and spatial variations in demand based on dynamic monitoring of the population. Furthermore, the varying accessibility of UGSs by multi-mode transport also suggests that planners should focus on the connectivity between UGSs and communities and draws a blueprint that is suitable for a multi-mode choice of transport. Finally, the social inequalities of UGS accessibility are explained by their historical and social context, although their associations are not always linear and the mechanism is complex (29, 57). Therefore, the inequalities are difficult to eliminate in the short term, but it is possible to adopt more flexible solutions.

### Policy Implications

This study identified that the Government should formulate equalizing policies to improve the accessibility of the disadvantaged groups, even though the growth rate of spatial accessibility did not stall or even reversed during the study period. Similar to the importance of balancing the relationship between urban development and social justice, community deprivation in urban green space access is also an urgent problem to be solved in current urban development. Sufficient provision and dynamic spatial optimization of public green spaces are essential to relieve the deprivation of disadvantaged groups. However, the work should not be limited to UGS planning. Previous studies have only provided limited advice rather than a well-established policy framework.

In the process, identifying the deprived communities with limited UGS accessibility is the prerequisite to resolving these issues. In the beginning, policymakers regularly analyzed the demographic changes through population dynamic monitoring. Recently, ubiquitous big data have been widely used in social science research, and social network applications that have check-in options, such as “microblogs” or Twitter, provide researchers with location-based data for studying user behavior when visiting UGSs. Nevertheless, as reflected in the user preferences on social networking, these social network applications are seldom used by disadvantaged groups. This means that many users who belong to a disadvantaged group or those without mobile phones are not included in the research. Big data could be used to supplement traditional sampling statistics to detect regions with poor accessibility. Some prior studies have demonstrated that high-SES groups have better access to public green spaces, partly owing to their greater ability to choose residences with high-quality living environments and participate in planning decisions relevant to their benefits (58). More efforts should be made to provide sufficient green spaces for those communities where disadvantaged groups are rapidly increasing. Ensuring green coverage must be made mandatory for areas with low-cost housing, including affordable and low-rent housing.

Additionally, governments and relevant departments should optimize the road network structure during urban construction. By targeting varying degrees of deprivation through multi-mode transport,they should promote planning and design principles through the “Narrow Roadways, High Density Network” to improve accessibility by public transport and strengthen the construction of cycling and pedestrian lane systems. Furthermore, shuttle busses that connect large-scale UGSs and deprived communities should be incorporated into the planning. The free bus transfer to large-scale UGSs based on existing subways can also be considered, particularly for disadvantaged groups.

Finally, a management system for differentiation and diversification of supply and the trade-off and compensation for UGSs needs to be established. Due to the heterogeneity of urban space and the inhabitants' characteristics, more flexible greening strategies are recommended to create better green space access and improve the inhabitants' daily recreation service experience. For example, for the limited accessibility of community-level UGSs in core urban areas, vertical greening, parkways, and greenways should be added to improve the diversity index and strengthen landscape connectivity (59), thus building a solid foundation for the 15-min physical exercise scale. As the urban periphery always has larger construction space, complete park systems should be established to meet residents' basic requirements. Additionally, the quality and service level of existing UGSs should be strengthened in both core urban areas or the urban periphery, and the diversified functions of UGSs should meet the needs of different groups, particularly older people in the context of an aging society. In public land management, the Government should avoid privatization or quasi-privatization of UGSs due to excessive concentration in high-SES residences. The additional value should be used as a special fund to invest in the construction of UGSs in underserved areas. In practice, policymakers should provide adequate and high-quality UGSs based on the principle of convenience and equality and according to the above-mentioned policy framework. In addition to building a foundation for the “Healthy China Initiative” by offering a convenient space for physical exercise, the UGSs around urban inhabitants should also be used to encourage the sharing of UGSs among all urban inhabitants.

## Data Availability Statement

The datasets for this article are not publicly available because of the institutional copyright issues. Requests to access the datasets should be directed to Shunqi Cheng, skylers@vip.qq.com.

## Author Contributions

All authors made a substantial, direct and intellectual contribution to the work and approved it for publication.

## Conflict of Interest

The authors declare that the research was conducted in the absence of any commercial or financial relationships that could be construed as a potential conflict of interest.

## Publisher's Note

All claims expressed in this article are solely those of the authors and do not necessarily represent those of their affiliated organizations, or those of the publisher, the editors and the reviewers. Any product that may be evaluated in this article, or claim that may be made by its manufacturer, is not guaranteed or endorsed by the publisher.
